# Progressive Nodular Histiocytosis: Report of a Case and Review of the Literature

**DOI:** 10.1155/2021/5531820

**Published:** 2021-09-17

**Authors:** Numbereye Numbere, Tatsiana Pukhalskaya, Blythe Bowman, Katelynn Campbell, Bruce Smoller

**Affiliations:** ^1^Department of Pathology and Laboratory Medicine, University of Rochester Medical Center, Rochester, New York, USA; ^2^Dermatology Group of Arkansas, Little Rock, Arkansas, USA; ^3^Department of Pathology, University of Arkansas for Medical Sciences, Little Rock, Arkansas, USA; ^4^Department of Pathology and Laboratory Medicine and Department of Dermatology, University of Rochester Medical Center, 601 Elmwood Ave, Box 626, Rochester, New York, USA

## Abstract

Progressive nodular histiocytosis (PNH) is a rare condition characterized by progressive eruption of multiple yellowish-brown papules and nodules on the skin and mucous membranes. We present the case of a 37-year-old Caucasian man with gradually increased appearance of nodular lesions on the forehead and right temple. These lesions were initially diagnosed as xanthomas and did not respond to intralesional injections of triamcinolone. Additional biopsy revealed an intense dermal infiltrate of foamy mononuclear epithelioid cells with a minor admixture of plasma cells, lymphocytes, and scattered multinucleated giant cells. On immunohistochemical staining, the lesional cells were positive for CD163 and CD68 and negative for CD1a, thus confirming a mononuclear-macrophage lineage. The clinical presentation and the histological impression lead to the diagnosis of PNH. This condition could be challenging, mimicking microscopically similar lesions of the non-Langerhans cell histiocytosis group. Although uncommon, PNH stands out due to its clinical and microscopic features and should be taken into consideration in the differential diagnosis of cutaneous histiocytoses.

## 1. Introduction

Progressive nodular histiocytosis (PNH) is a rare normolipemic cutaneous xanthogranulomatous disorder that belongs to the group of mucocutaneous non-Langerhans cell histiocytoses [1]. Clinically, PNH is characterized by progressive eruption of multiple yellow-brown papules and nodules on the skin and mucous membranes [2, 3]. Histologically, foamy tissue macrophages and spindle-shaped cells within a fibrocollagenous matrix may be seen [4]. Touton cells may also be present. Although uncommon, accurate diagnosis of PNH is important because of its persistent and progressive clinical course.

## 2. Case Presentation

A 37-year-old Caucasian male presented with a history of multiple persistent lesions on his forehead and right temple. His past medical history was essentially noncontributory and included premature atrial contractions controlled with propafenone and escitalopram. His labs showed normal triglycerides and borderline elevated cholesterol (total 5.41 mmol/L; LDL 3.38 mmol/L).

**Figure 1:**
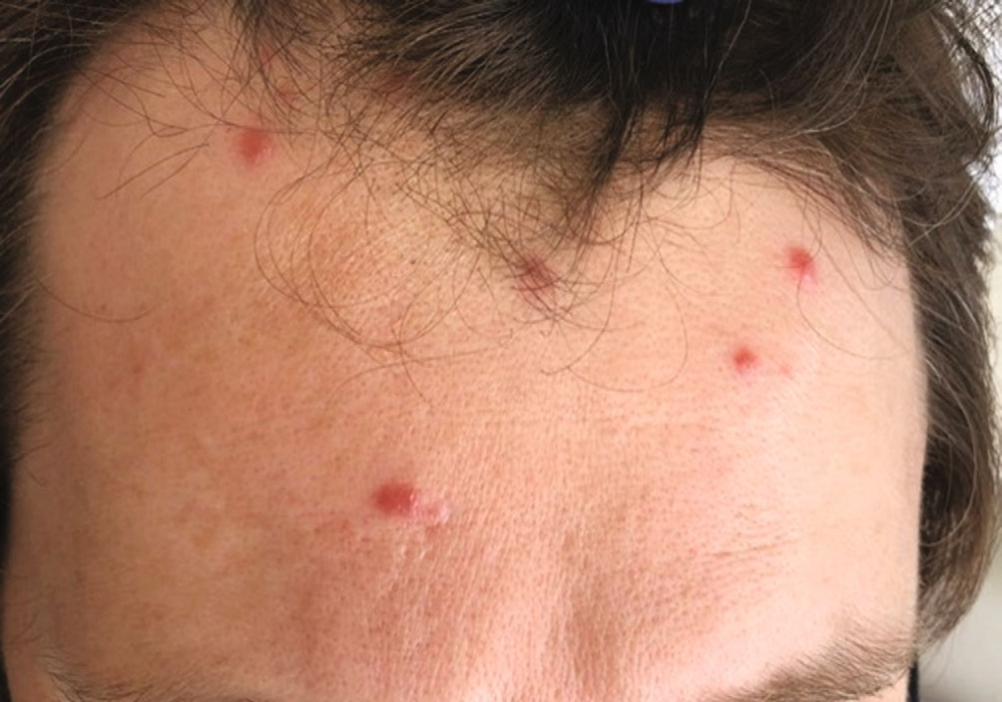
Clinical photograph showing multiple nodules on the patient's forehead.

The patient initially reported 2-3 lesions that gradually increased in number in the following months. All lesions looked identical and described as moderately painful pink violaceous papules and nodules with irregular borders (Figure 1). The initial biopsy revealed a prominent xanthogranulomatous infiltrate in the dermis and was diagnosed as a xanthoma. The lesions were treated unsuccessfully with intralesional triamcinolone, and the presence of increasing numbers of lesions resulted in the need for further histologic evaluation.

**Figure 2:**
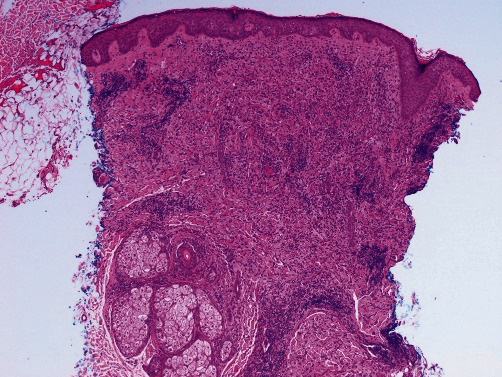
Micrograph of the forehead lesions demonstrating intense dermal-based infiltrate of foamy macrophages with sparing of skin adnexa. H&E ×40.

**Figure 3:**
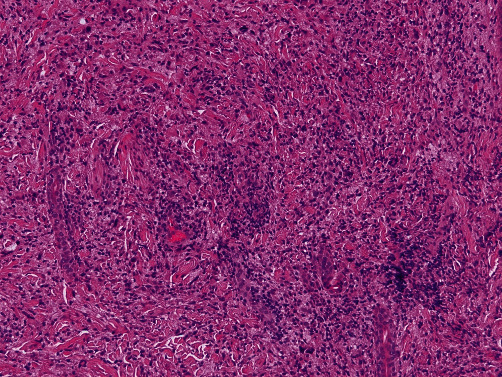
Higher-power view revealed predominantly foamy tissue macrophages with a minor admixture of plasma cells, lymphocytes, and scattered multinucleated giant cells. H&E ×100.

**Figure 4:**
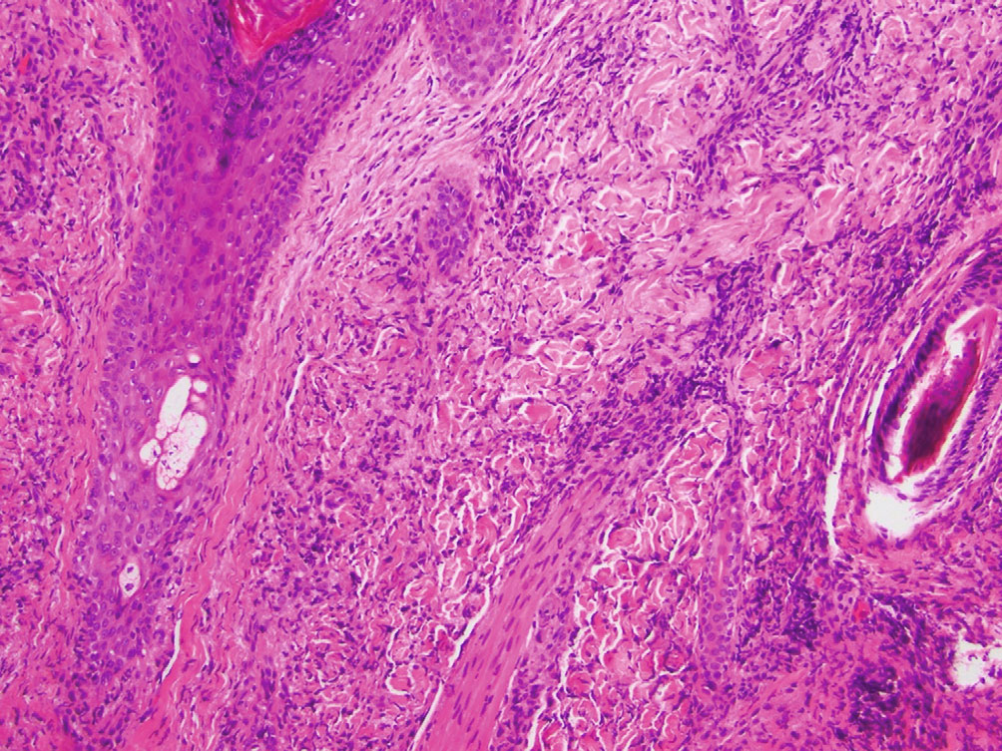
A close up of a different field shows a predominance of spindled cells with prominent extracellular collagen deposition.

**Figure 5:**
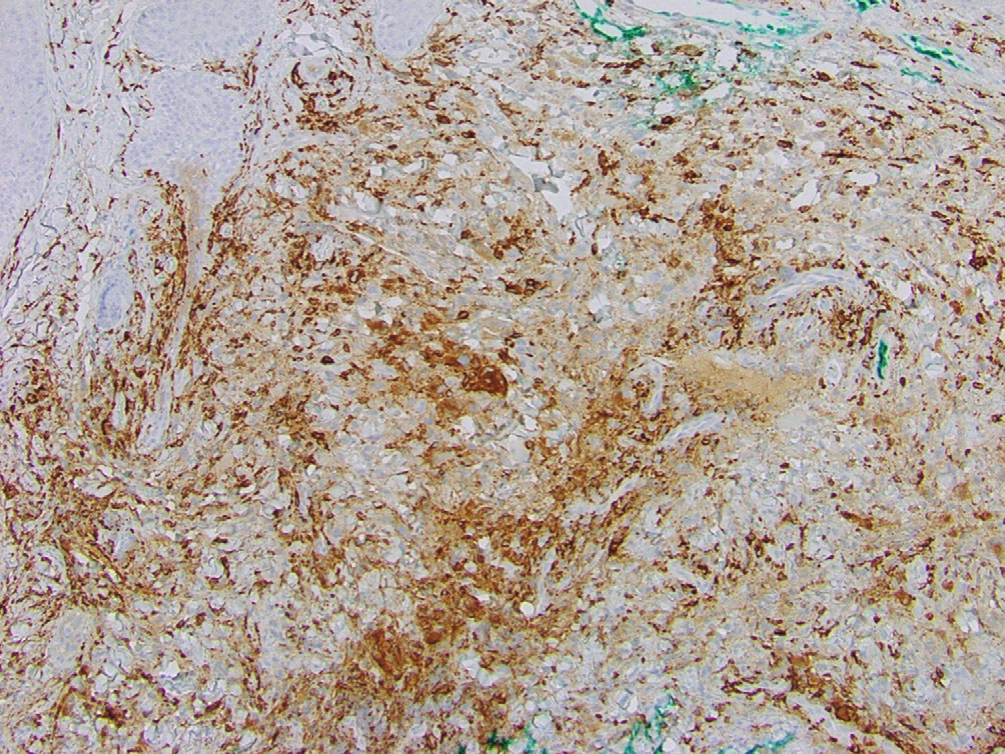
CD163 immunohistochemical study highlights the tissue macrophages. ×100 magnification.

Histologic evaluation of the recurrent/persistent lesions revealed an intense dermal infiltrate of foamy tissue macrophages with a minor admixture of plasma cells, lymphocytes, and scattered multinucleated giant cells (see Figures 2 and 3). Other areas showed a predominance of spindled cells and associated extracellular collagen fibers (see Figure 4). The nuclei of lesional cells did not exhibit hyperchromasia or pleomorphism, and no mitotic figures were seen. Adjacent skin adnexa were spared. The lesional cells were positive for CD163 (see Figure 5) and CD68 and negative for CD1a, thus confirming them to be of mononuclear-macrophage lineage. The impression on microscopy interpreted alongside the clinical presentation of multiple nodules and the patient's essentially normal serum lipids narrowed the diagnosis to PNH.

## 3. Discussion

Since Taunton et al. described the first case of progressive nodular histiocytoma (subsequently renamed progressive nodular histiocytosis) in 1978 [3], our knowledge of this rare entity has been largely limited to information gleaned from a few case reports or small case series. PNH is a noncongenital, nonfamilial, and nonlipemic mucocutaneous proliferative disorder of macrophages. The etiology is unknown. A cytogenetic study of lesional cells showed a normal karyotype, possibly signifying a nonneoplastic etiology [5].

PNH most commonly affects young to middle-aged adults who present with nonpainful, nonpruritic, widely disseminated, randomly distributed red-brown cutaneous papules and nodules [6]. A gender predilection has not been reported, and there is no syndromic association. The clinical course of PNH is characteristically relentless without spontaneous remission. Lesions increase in number and size over time and can be markedly disfiguring [7–10]. Mucosal involvement may occur [9, 10], but internal organs are not usually affected [10]. Functional impairment may occur from mechanical interference brought on by critically positioned lesions on sites like the soles of the feet and eyelids [10, 11]. On very rare occasions, the cutaneous lesions may directly invoke systemic effects—a case of microcytic anemia from marked intralesional iron sequestration is on record [10]. Although not usually life-threatening, death has been reported from obstructive lesions in the upper airway [11].

The superficial papules, deep nodules, and mucosal polyps of PNH span a histologic continuum. The superficial lesions show a diffuse infiltrate of foamy tissue macrophages. Fibrosis in the deeper lesions imparts a spindled appearance to the lesional cells, which are often arranged in a storiform pattern. Scattered multinucleated Touton giant cells and a modest infiltrate of lymphocytes and plasma cells are encountered in both types of lesions. The lesions of PNH may show focal infiltration of proximate structures like skeletal muscle, but nuclear/cytologic pleomorphism, hyperchromasia, necrosis, mitotic figures, or other features of malignancy are characteristically absent [9]. The lesional tissue macrophages are immunoreactive with CD163, CD68, and factor XIIIa. In contrast to Langerhans cell histiocytosis (LCH), they do not mark with S100 protein, CD1a [10], or CD207/langerin.

The various mucocutaneous non-LCH histiocytoses share some light microscopic features [1, 12, 13]; thus, correlation of the clinical, histopathologic, and immunohistologic features is needed to clearly distinguish PNH. Benign cephalic histiocytosis (BCH) and juvenile xanthogranuloma (JXG) are afflictions of childhood [12]. Solitary reticulohistiocytoma (SRH) and classic cases of JXG and adult xanthogranuloma (AXG) are solitary, nonprogressive lesions in contradistinction to the scores of continuously emerging lesions that are typical of PNH [14, 15]. However, multifocal cutaneous lesions in JXG, AXG, and generalized eruptive histiocytosis (GEH) mimic the clinical picture of PNH to a greater degree [16–20]. Prominent mucosal and visceral involvement in addition to a well-characterized association with diabetes insipidus (50% of cases) in xanthoma disseminatum (XD) help to distinguish it from PNH [21, 22]. Up to 10% of patients with JXG may present with internal organ involvement [23], but visceral involvement in PNH is a distinct departure from the norm. Unlike PNH, GEH and BCH frequently undergo spontaneous resolution [24–28]. The distinctive oncocytic cells that characterize SRH and multicentric reticulohistiocytosis are not seen in PNH [29, 30].

Elevated serum/plasma lipid levels help to differentiate PNH from eruptive histiocytoses that sometimes occur in association with hyperlipidemia [31]. A careful search for microorganisms, aided by the requisite histochemical or immunohistochemical stains, is needed to rule out eruptive forms of parasitic, fungal, or mycobacterial infections [32]. Due to its varied morphologic manifestations, the microscopic findings of dermatofibroma (DF)/benign fibrous histiocytoma (BFH) may mimic PNH [33, 34]. However, DF/BFH are most commonly solitary, nonprogressive lesions and are thus lower in the clinical differential diagnosis. Owing to its predominant cutaneous manifestation, detailed systemic workup (other than perhaps a lipid profile) is usually not required for diagnosis or management, unless indicated by the patient's symptoms [8].

Ever so often, the clinicopathologic features of histiocytoses manifest “shades of gray” rather than clear-cut clarity. Lesions with overlapping features have been described. Entities with JXG and PNH overlap are on record [9, 35], as are lesions with clinicopathologic features that bridge PNH and multiple AXG [12]. There have also been well-documented cases of transformation of one entity into another, e.g., BCH evolving to JXG [36] and GEH morphing into XD, JXG, or PNH [37]. Some authors regard GEH not as a discrete entity, but as the early stage of various mucocutaneous histiocytoses including PNH [20, 38]. Sometimes, these lesions evade precise nosologic categorization, and the best that can be proffered even after a thorough evaluation of all details of a case is a list of competing possibilities. Our diagnosis of PNH is based on the patient's clinical presentation with persistent and increasing number of lesions, supported by the aforementioned histologic findings. The clinicopathologic features of PNH and the other entities in the differential diagnosis are compared in Table 1.

Surgical excision remains the mainstay of treatment of PNH [9, 10]. But there have been a few reports of improvement after administration of methotrexate [46]. PNH has largely proven resistant to other treatments like intralesional and systemic steroids [10], carbon dioxide laser, and antineoplastic agents like imatinib [6]. Unfortunately for afflicted patients, recurrence may occur after treatment [9].

## 4. Conclusion

In summary, we present the case of a 37-year-old man with sudden onset of multiple cutaneous papules and nodules of PNH. The diagnosis of PNH is greatly dependent on clinicopathologic correlation. The unrelenting nature of the disease and its resistance to therapy makes it critical to distinguish PNH from several close mimics that, in many cases, follow a less relentless clinical course.

## Figures and Tables

**Table 1:** Comparison of clinicopathologic features of the different entities in the differential diagnosis of progressive nodular histiocytosis.

Clinicopathologic feature	PNH	BCH [25]	JXG [23]	AXG [39]	GEH [26]	XD [28]	SRH/MRH [40]	Dermatofibroma [41]
Age at onset	Middle-aged to older adults	Typically in the first year of life	Childhood	Adulthood	Adults and children can be affected	Most before 25 years of age	Median age 35 years	Most common in the 20s to 40s

Pattern of cutaneous manifestation	Multiple, widespread cutaneous papules and nodules	Multiple papules most commonly on the head and neck	Solitary cutaneous or subcutaneous nodules in up to 83% of cases	Solitary nodule in up two-thirds of cases	Dozens to hundreds of papules over the trunk and extremities	Multiple and widespread papules	Cutaneous papule(s) or nodule(s)	Usually solitary with predilection for the extremities

Mucous membrane involvement	Yes	Rare	Yes	No	Rare	Yes	No (SRH); yes (MRH)	Yes (benign fibrous histiocytoma) [42]

Visceral involvement	Not usual	No	Yes, in up to 10% of cases	Not usual	Not usual	Yes	No (SRH); yes (MRH)	No

Association with systemic disease	No	Not usual [43]	Can occur	No	Not usual	Yes, 50% of cases associated with diabetes insipidus	MRH may be associated with systemic vasculitis [44] and malignancy [45]	No

Histology	Spindled lesional cells in the dermis	Demarcated infiltrates of tissue macrophages in the reticular dermis	Foamy and spindled mononuclear cells, Touton giant cells	Foamy and spindled mononuclear cells, Touton giant cells	Dermal collection of spindled cells and scattered Langhans giant cells	Macrophages with scalloped nuclei and foamy cytoplasm, as well as Touton and foreign body-type giant cells	Circumscribed aggregate(s) of oncocytic epithelioid cells in the upper and middermis	Poorly circumscribed dermal proliferation of spindled and epithelioid cells

Clinical course	Usually progressive and unremitting	Frequently undergoes spontaneous regression	Spontaneous regression is common in children but less so in adults [39]	Spontaneous regression is uncommon	Frequently undergoes spontaneous regression	Most frequently persistent; spontaneous regression is rare	Does not undergo spontaneous regression	Spontaneous regression is uncommon

Recurrence	Yes	No	No	No	No	No	No	No

PNH: progressive nodular histiocytosis; BCH: benign cephalic histiocytosis; JXG: juvenile xanthogranuloma; AXG: adult xanthogranuloma; GEH: generalized eruptive histiocytosis; XD: xanthoma disseminatum; SRH: solitary reticulohistiocytosis; MRH: multicentric reticulohistiocytosis.
